# Optimization of the Centrifugal Spinning Parameters to Prepare Poly(butylene succinate) Nanofibers Mats for Aerosol Filter Applications

**DOI:** 10.3390/nano13243150

**Published:** 2023-12-15

**Authors:** Ayben Pakolpakçıl, Ali Kılıç, Zbigniew Draczynski

**Affiliations:** 1 Faculty of Textile Technologies and Design, İstanbul Technical University, İnönü Cad, No 65 Gümüşsuyu, Beyoğlu, 34421 Istanbul, Türkiye; alikilic@itu.edu.tr; 2 Faculty of Art and Design, İstanbul Nişantaşı University, Maslak Mahallesi, Taşyoncası Sok, No 1V-1Y, Sarıyer, 34398 Istanbul, Türkiye; 3 Institute of Materials Science of Textiles and Polymer Composites, Lodz University of Technology, 116 Zeromskiego Street, 90-924 Lodz, Poland; zbigniew.draczynski@p.lodz.pl

**Keywords:** centrifugal spinning, poly(butylene succinate), nanofiber, filter, response surface methodology, Box–Behnken

## Abstract

Air pollution is becoming a serious issue because it negatively impacts the quality of life. One of the first most useful self-defense approaches against air pollution are face masks. Typically made of non-renewable petroleum-based polymers, these masks are harmful to the environment, and they are mostly disposable. Poly(butylene succinate) (PBS) is regarded as one of the most promising materials because of its exceptional processability and regulated biodegradability in a range of applications. In this regard, nanofiber-based face masks are becoming more and more popular because of their small pores, light weight, and excellent filtration capabilities. Centrifugal spinning (CS) provides an alternative method for producing nanofibers from various materials at high speeds and low costs. This current study aimed to investigate the effect of processing parameters on the resultant PBS fiber morphology. Following that, the usability of PBS nonwoven as a filter media was investigated. The effects of solution concentration, rotating speed, and needle size have been examined using a three-factorial Box–Behnken experimental design. The results revealed that PBS concentration had a substantial influence on fiber diameter, with a minimum fiber diameter of 172 nm attained under optimum production conditions compared to the anticipated values of 166 nm. It has been demonstrated that the desired function and the Box–Behnken design are useful instruments for predicting the process parameters involved in the production of PBS nanofibers. PBS filters can achieve an excellent efficiency of more than 98% with a pressure drop of 238 Pa at a flow rate of 85 L/min. The disposable PBS filter media was able to return to nature after use via hydrolysis processes. The speed and cost-effectiveness of the CS process, as well as the environmentally benign characteristics of the PBS polymer, may all contribute considerably to the development of new-age filters.

## 1. Introduction

Air pollution is a substantial concern to human health, owing mostly to the emission of fine particulate matter (PM) into the atmosphere by cars and industrial processes. It has been established that exposure to outdoor particles has adverse effects on health. The considerable rise in the infectivity and mortality of the coronavirus disease has been directly attributed to even a slight increase in air pollution after the onset of the infectious severe acute respiratory coronavirus 2 (SARS-CoV2) sickness. According to a United Nations Economic Commission for Europe (UNECE) report, air pollution destroys seven million people each year, which is roughly four times the death rate from the human immunodeficiency virus (HIV) and nearly six times the death rate from malaria. Fine particles can easily penetrate the lungs and bronchi. They have the potential to create severe health problems, including respiratory, cardiovascular, and even carcinogenic disorders, according to the World Health Organization (WHO) [1,2]. Face masks are used as the main personal protective equipment (PPE) to protect against the inhalation of particulates [3]. Indeed, there is a significant increase in the consumption of face masks, which have witnessed a significant increase in manufacture throughout the pandemic era due to increased air pollution. Facemasks are primarily made of nonbiodegradable polymers such as polypropylene (PP), polyurethane (PU), polyethylene (PE), and their disposal generates a significant amount of waste, producing serious environmental difficulties [4,5,6]. Because of global pollution concerns, several standards for future advanced materials have altered, and it is now vital to create sustainable items while simultaneously addressing the biodegradability and renewability of the materials used. Polycondensing 1,4-butanediol with succinic acid results in the bioplastic poly(butylene succinate) (PBS). Succinic acid may be generated by microbial fermentation from renewable feedstocks such as starch, sucrose, or glucose. It’s an aliphatic polyester that is flexible, melt-processable, and chemically resistant. It is frequently used and discarded in everyday life and possesses properties such as PE and PP. It may also be mixed with other bioplastics to improve its properties before being used in packaging, biodegradable bags, and mulching films. PBS is therefore a polymer that has the potential to replace synthetic plastics while also minimizing the accumulation of plastic trash. PBS can be broken down into water and carbon dioxide via hydrolysis or enzymatic oxidation [7,8,9].

Nanofibers are produced using several techniques, including electrospinning, rotary jet spinning, solution blowing, and melt blowing. Numerous experts have observed that as the fiber diameter is reduced, more surface area becomes accessible, and as a result, the filtering performance may increase. Furthermore, a lower fiber size improves the capture effectiveness, especially for particles with a diameter of 300 nm or less. As a result, using nanofiber materials in filter applications is highly beneficial [10,11]. PBS nanofiber is generated by an electrospinning process because it is a low-cost and straightforward method of producing nanomaterial [12,13,14,15,16,17,18,19,20,21]. However, the production rate of electrospinning is limited, with flow rates ranging from 0.5 mL/h to 300 nL/min. A little sample of polymeric fiber might take hours to collect, which can cause problems with needle blockage that delays the procedure.

The centrifugal spinning (CS) method is used to manufacture fibers by applying high rotation to a solution within a cylinder having one or more holes. Consequently, the polymer is stretched when it leaves the main cylinder as the fluid is forced (by centrifugal force) through these holes. Along the transit between the cylinder and the bulkhead, the liquid evaporates the solvent or lowers the temperature for solidification, eventually reaching the bulkhead and depositing polymeric fibers. CS has several advantages. For example, it is simple to use and does not need any electric fields or potential differences, it may be used for polymer melts or concentrated solutions. As long as the viscosity is appropriate, it still provides good productivity with low price [22,23]. A variety of polymers, such as poly(3-hydroxybutyrate-co-3-hydroxyvalerate) (PHBV) [24], poly(acrylonitrile) (PAN) [25], polystyrene (PS) [26,27], poly(methyl methacrylate) (PMMA) [28], poly(lactide) (PLA) [29,30], poly(ethylene oxide) (PEO) [31], poly(ε-caprolactone) (PCL) [32], poly(vinyl alcohol) (PVA) [33], (poly(vinyl pyrrolidone) (PVP) [34], poly(vinylidene difluoride) (PVDF) [35], poly(ethylene terephthalate) (PET) [36], polyamide 6 (PA6) [37], polyurethane (PU) [38], polyamic acid (PAA) [39], hyaluronic acid (HA) [40], chitosan [41], lignin [42], silk fibroin [43] and gelatin [44] are fabricated by CS method. Fiber manufacturing using the CS technique with a PBS polymer has not been studied. Hence, the objective of this present work is to optimize the process parameters of the CS method on a PBS nanofiber and, then investigate its suitability as a filter.

Herein, PBS successfully developed a highly efficient degradable filter using the centrifugal spinning process. Analyses of the CS process parameters, including the rotating speed, needle size, and solution concentration, were conducted to identify the low-diameter, bead-free nanofiber. To identify the ideal CS processing parameters to maximize the formation of PBS nanofibers, a three-factor three-level Box–Behnken design was employed. To the best of our knowledge, no studies on the PBS nanofiber for filtering via CS technique have been published. Independent variables’ effects on fiber diameter were investigated. The filtering performance as well as several physical features have been studied. The PBS nanofiber achieved a high efficiency of 98% with a pressure drop of 238 Pa at a face velocity of 14.17 cm/s, meeting the NIOSH 42 CFR Part 84 standard (N95 certification norms, efficiency > 95% and pressure drop 343 Pa) [45]. This study could be able to recommend a simple and quick technique for producing air filters that are economical and highly effective for use in facemasks.

## 2. Experimental Study

### 2.1. Production of PBS Fiber

**Figure 1:**
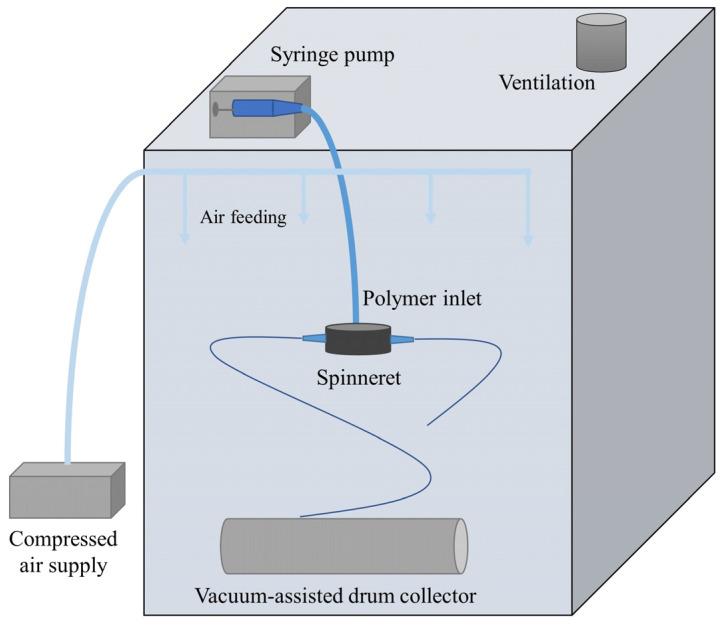
The schematic diagram of the centrifugal spinning machine.

PBS pellets (density 1.36 g/cm^3^ and melt flow index MFI [190 °C, 2.16 kg], 22 g/10 min) were purchased from a company (PTT MCC Biochem Company Ltd., Bangkok, Thailand). The solvents employed in this study were chloroform and ethanol from Merck & Co., Darmstadt, Germany, both of which were used exactly as received. PBS solutions with concentrations of 7, 10, and 13 wt.% were produced by dissolving in a mix of chloroform and ethanol 3:1 (*v*/*v*) [14] and continuously stirring at 400 rpm (ISOLAB GmbH, Wertheim, Germany) for 10 h. The PBS nanofibers were produced using the provided solutions in a lab-scale centrifugal spinning machine (NanoCentrino, AREKA Group Ltd., İstanbul, Turkey). The schematic diagram of the centrifugal spinning machine is given in Figure 1. A spinneret, orifices, and needles are constituents of the system. The needles are inserted into the orifices. The spinneret is powered by a high-speed motor. A scalable vacuum pump drives a spinning collector, which gathers fibers uniformly on its surface.

Three of the most vital variables involved with fiber development are chosen as design factors. Table 1 shows an orthogonal design with three layers created using the Box approach. For each sample, 10 min productions were carried out. The PBS fiber was collected on the nonwovens with the help of a vacuum-assisted, rotating collector (circumference = 40 cm and width = 40 cm). The needle-to-collector distance was set to 26 cm, and the collector speed was set to 1500 rpm. All solution preparations and spinning processes were carried out at room conditions (temperature = 25 ± 2 °C; relative humidity = 45 ± 5%).

### 2.2. Characterizations and Measurements

The morphological appearances and diameters of the PBS fiber were examined using a scanning electron microscope (SEM, Tescan Vega 3). The average fiber diameters are shown by 50 randomly selected fibers from the entire region of the SEM images. The results were presented as the mean standard deviation.

The filtration efficiency and filter resistance of the PBS filter were tested using an automated filter tester (TSI Model 8130, Inc., Shoreview, MN, USA). Particles of sodium chloride (NaCl) with a diameter of 0.26 mm that were charge-neutralized and monodisperse solid was used. The device has a 100 cm^2^ sample measurement area. Measurements were repeated three times. The results were displayed as the average ± standard deviation. The ratio of collected to input particles was used to determine each sample’s filtering efficiency. In the same way, each sample’s pressure drop was measured. The equation for calculating filtration effectiveness (η) is η = (C_i_ − C_f_)/C_i_, where C_i_ and C_f_ represent the beginning and final concentrations of pollutants, respectively. Using these parameters, the quality factor (QF) values were calculated. QF = −ln (1 − η)/ΔP, where ΔP and η represent the nonwoven’s filtering efficiency and pressure drop, respectively, was used to calculate the QF.

The surface area, cumulative pore volume, and average pore diameter of the PBS filter were determined using the NOVA touch 4LX (Quantachrome TouchWin Version 1.21, Boynton Beach, FL, USA) with low-temperature (77 K) nitrogen adsorption isotherms recorded across a wide range of relative pressures from 0.00 to 1.00. Each sample was degassed for 3 h at 100 °C before being measured. The surface area of the samples was measured using the multipoint Brauner–Emmet–Teller (BET) technique. Barrett–Joyner–Halenda (BJH) was utilized to measure cumulative pore volume.

A goniometer (Theta-lite, Biolin Scientific, Västra Frölunda, Sweden) was used to measure the water contact angle of the PBS filter. Distilled water was dropped onto the sample. An evaluation program (Oneattension, Biolin Scientific, Sweden) was used to take and analyze the photos. The average of the measurements obtained from the two sides of each water droplet was used to record the data for each measurement. Measurements were repeated 3 times. The results were displayed as the average ± standard deviation.

The PBS filter’s mechanical characteristics were assessed using an apparatus (Instron 5944, Norwood, MA, USA). The crosshead speed was 10 mm/min, the starting grasp separation was 50 mm, and the nanofibrous mats were cut into rectangular samples (10 × 2 cm^2^). Prior to measurement, the samples had been preserved for 24 h in a conditioned setting with 20 ± 2 °C and 65 ± 4% humidity. They were subsequently analyzed in the same setting. The results were displayed as the average ± standard deviation.

Ten points on the PBS filter were measured for thickness using a digital thickness gauge (Loyka 5318, Loyka Instruments, İstanbul, Turkey).

The PBS filter was cut into approximately 2 × 2 cm^2^ and weighed. At room temperature, the PBS filter was soaked in 1N of NaOH solution for 3, 6, 12, and 24 h, respectively. After that, the samples were collected and washed with distilled water before being dried in a vacuum oven at 50 °C. Weight loss determines the degree of deterioration [21]. The equation for degradation degree (%) = (W_0_ − W_t_)/W_0_ × 100 was used, where W_0_ is the starting weight of PBS nanofiber and W_t_ is the weight of PBS fibers after hydrolysis.

### 2.3. Statistical Analysis

Response surface methodology (RSM) is a statistical method used in developing and improving the quality characteristics of a product or process. RSM involves creating a mathematical model that best fits the data obtained by using an experimental design that ensures an adequate and reliable measurement of the response variable with a minimum number of observable values and determining the factor levels that give the best response value. Many experimental designs can be made with RSM. However, designs such as Box–Behnken and central composite experimental designs, which were specially developed for RSM and significantly reduce the number of experiments, are widely used [45,46]. Models such as linear, quadratic and cubic are used with RSM. The equation of the quadratic model can be expressed as in Equation (1).

(1) $$Y=\beta_{0}+\sum_{j=1}^{k}\beta_{i}x_{j}+\sum_{j=1}^{k}\beta_{ij}x_{j}^{2}+\sum_{i}^{k-1}0\sum_{j}^{j}\beta_{ij}x_{i}x_{j}+\varepsilon$$

In this equation, the independent variables *x*_1_, *x*_2_, …, *x*_*n*_, the dependent (response) variable *y*, *β*_0_ *β_i_*, *β_ii_* (*i* = 1, 2, …, *n*), *β_ij_* (*i* = 1, 2, …, *n*; *j* = 1, 2, …, *n*) denote the unknown model parameters and *ε* the random error term.

In this research, a 3 × 3 Box–Behnken design (BBD) was employed to optimize the independent variables with the software (Design Expert 13, Minneapolis, MN, USA). According to the literature, the solution concentration, rotating speed, and nozzle size have more impact on fiber diameters than other factors [23]. Thus, these features were chosen as independent variables, with the average fiber diameter acting as the response value. Table 2 shows how they were modified at three levels: low (−1), middle (0), and high (+1). The BBD technique has 15 test runs. The statistical significance of the predicted model was determined using the analysis of variance (ANOVA) and least squares methods. Degrees of freedom (df), the sum of squares (SS), and mean squares (SM) are indicated in ANOVA table. The variable’s mean squared error (MS) is divided by the error’s mean square error (MS) to determine the F value. The region under the proper null sampling distribution of F that is larger than the observed F-statistic is the ρ-value.

## 3. Results and Discussion

### 3.1. Model Development

The CS process is examined in the current study using RSM in conjunction with BBD, and Table 1 displays the findings. The sum of squares for the CS process in a sequential mode is displayed in Table 3. The model of the CS process’s summary statistics is displayed in Table 4. Below is an equation with coded factors.

**Figure 2:**
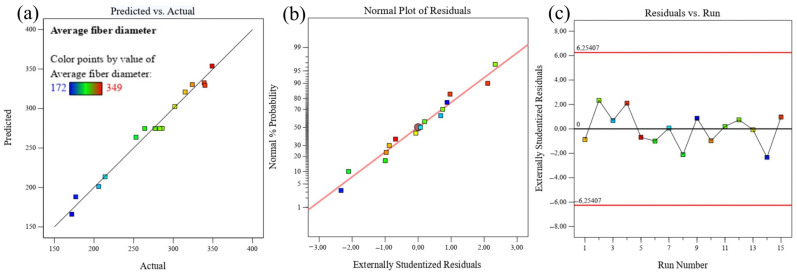
(**a**) Predicted vs. actual nanofiber sizes (**b**) Normal probability, and (**c**) Residual plots.

Y = 274.66666666667 + 60.375A + 17B + 15.875C + 6AB + 9.75AC + 17.5BC + 41.708333333333A^2^ + 4.5416666666667B^2^ + 34.791666666667C^2^ (2)

where Y, A, B, and C represent fiber diameter, concentration, rotating speed, and needle size. The quadratic model’s ANOVA results in Table 5 demonstrated that the model equation could be applied to the CS process under a broad range of producing parameters. Table 5 shows that when the F-value is greater than 20, the quadratic model is highly significant at the 95% confidence interval. ρ-values less than 0.0500 indicate the significance of the model terms. The model terms A, B, C, BC, A^2^, and C^2^ are important in this situation. It is implied that the model terms are unimportant by values greater than 0.1000. In the current investigation, an appropriate accuracy value of >4 is found, indicating a strong connection between the actual and projected values. Figure 2a depicts a plot of the actual and expected nanofiber sizes, Equation (2). The data points on this plot are relatively near to the straight line, indicating that the developed model is suitable for representing the CS process and producing the necessary nanofiber diameter.

The average diameter of PBS nanofibers ranged from 172 to 349 nm depending on the CS parameters. Other researchers have described in the literature the production of various different-sized PBS of 125–315 nm [12], 1 µm [14], 292–454 nm [15], 480–743 nm [16], 0.43–4.20 μm [17], 4.16 μm [18], 400–430 nm [19], and 290–640 nm [20] via electrospinning.

The percentage of total variability described by the regression model is represented by the measure of goodness of fit, or R^2^ [47]. The average fiber diameter variability can be explained by the model in 98% of cases, as indicated by the R^2^ value, which is around 0.98210. The adjusted R^2^ of 0.9499 and the predicted R^2^ of 0.7680 are reasonably in agreement; the difference is less than 0.2.

The model validity may be examined visually using residual plots (Figure 2). The discrepancy between an observed value and an estimated value is referred to as the residual. In the case of a normal distribution, the residuals with the anticipated value are shown using normal probability plots (Figure 2b). According to the graph, the residuals appear to linearly correlate, which is a sign that the mistakes are distributed regularly. A residuals vs. observation order graph (Figure 2c) also looks at the question of whether the residuals are independent of the order in which the data are observed. The residuals on the graph often exhibited a random pattern, which suggested that the observational order had no bearing on the outcome and that the residuals were unrelated.

### 3.2. The Effect of the CS Procedure on PBS Nanofiber

**Figure 3:**
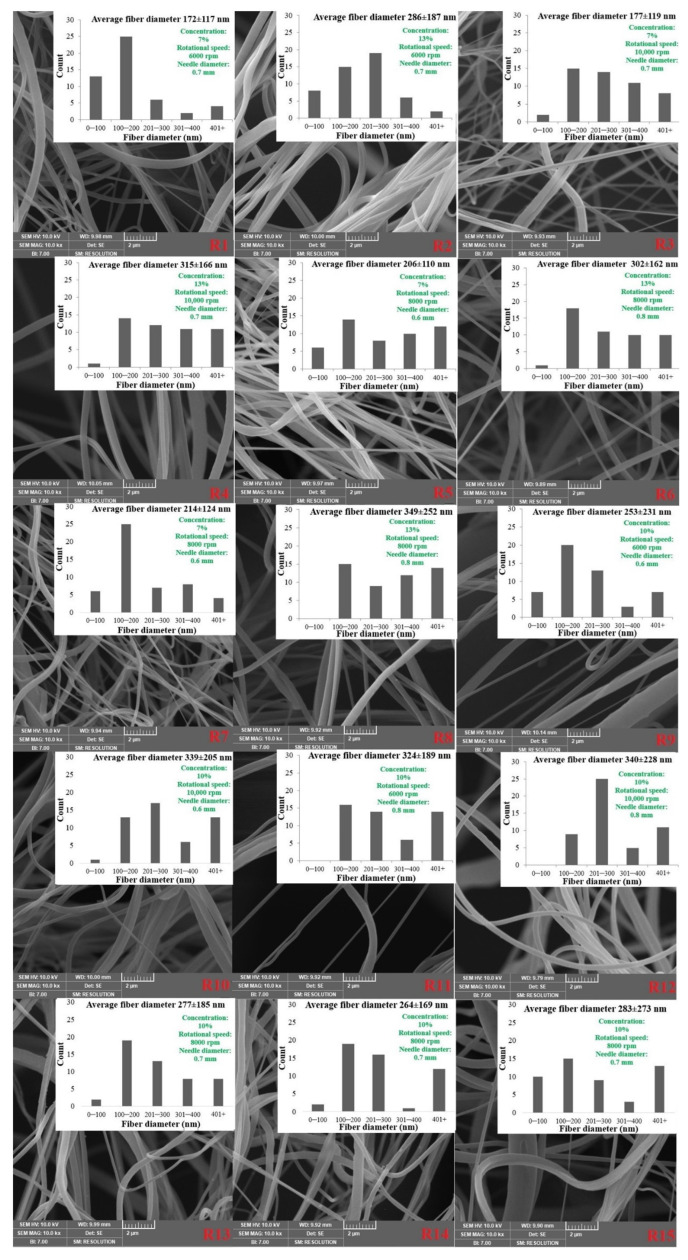
SEM images and fiber diameter distribution of PBS fibers (R1–R15) in Table 1.

**Figure 4:**
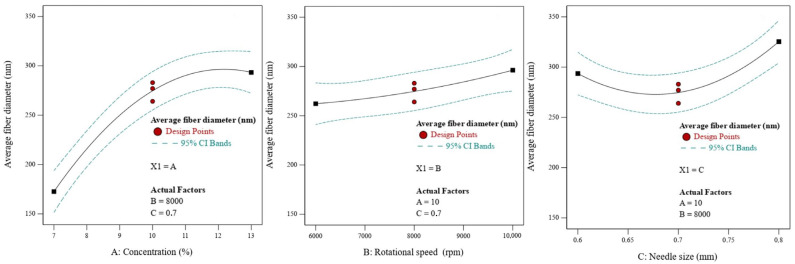
The main effects for average fiber diameter of PBS fiber diameter.

The literature has demonstrated how the CS process is dependent on factors like solution concentration. [35,48,49]. The influence of solution concentration on the structure of the nanofiber and the CS process has been investigated by using different concentrations, as indicated in the experimental section, to create PBS nanofiber. Figure 3 displays SEM images of the PBS nonwovens. The PBS nanofiber showed randomly oriented fibers, as seen by SEM pictures. Figure 4 illustrates how the diameters of the fibers increased from around 170 nm to 350 nm as the concentration increased from 7% to 13%. This is mostly because of the increased spinning solution concentration and the proportion of polymer that accompanied it [27]. These factors improved the cohesion and entanglement between the macromolecular chains, which helped to promote the growth of fiber diameter and smoothness of the surface.

Another significant factor influencing the average fiber diameter and shape is rotational speed [23,49]. By changing the rotational speed from 6000 rpm to 10,000 rpm, the effect of the rotational speed was examined. The diameters of the fibers increased from around 260 nm to 290 nm as the rotating rates increased from 6000 rpm to 10,000 rpm, as seen in Figure 4. The fiber diameter increased as a result of the faster rotation speed, shorter shot time, and faster fiber traveling to the receiving device.

Nozzle diameter is one of the other operational elements that might affect fiber morphology. By regulating the mass throughput of the liquid jet, altering the nozzle size can alternate the nanofiber structure without directly impacting the centrifugal force applied to the solution jet [49]. The average fiber diameter decreased from around 290 to 270 nm when the nozzle size was increased from 0.6 to 0.7 mm. It is assumed that at the small needle size, clogging in the polymer solutions might occur. Hence, it was found that the fiber diameter increased when 0.6 mm needle size was used. The average fiber diameter rose from around 270 to 320 nm when the nozzle size was increased from 0.7 to 0.8 mm (Figure 4). Higher mass outcomes and higher fiber diameter are produced by increasing the nozzle diameter [50].

**Figure 5:**
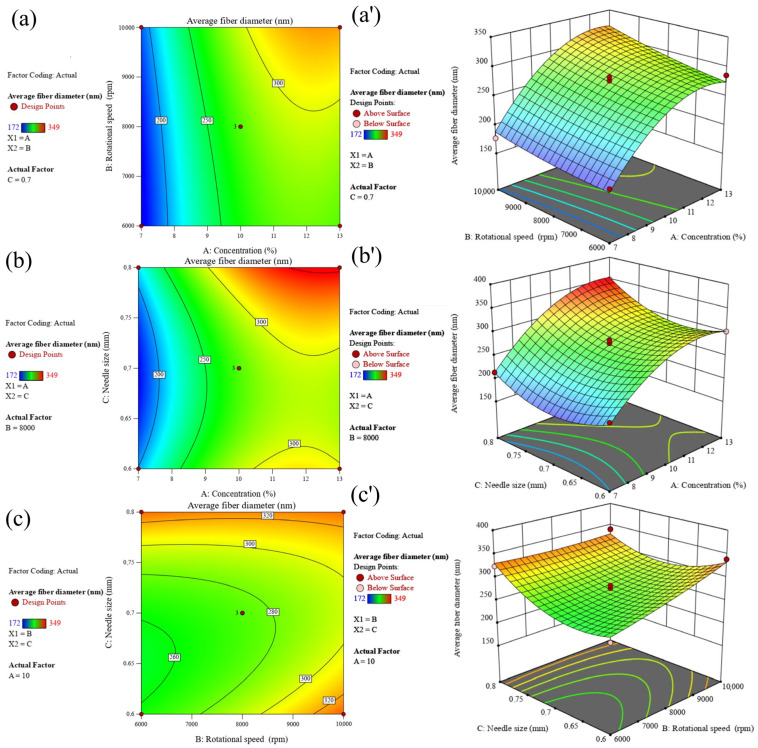
2D–3D response surface plots of PBS diameter versus (**a**,**a′**) concentration and rotational speed, (**b**,**b′**) concentration, and nozzle size, and (**c**,**c′**) rotational speed and nozzle size.

The interaction of the relevant components was plotted in this study using Design-Expert version 13 software to illustrate the impacts of solution concentration, rotational speed, and nozzle size on the diameter of nanofiber. The response variable (average fiber diameter) as a function of the chosen factors (two factors at a time) was plotted in three dimensions (3D) as shown in Figure 5a–c. The response values for PBS diameter are better illustrated by the color representation, where greater fiber diameters are displayed in red and lower fiber diameters in blue.

### 3.3. Investigation of PBS Nanofiber as a Filter

Particulate matter (PM) is a hazardous solid and liquid droplets found in contaminated surroundings that are released into the air [51]. PM_0.3_ (≤0.3 µm), PM_2_._5_ (≤2.5 µm), and PM_10_ (≤10 µm) are among the aerosols that degrade the environment. All these aerosols, PM_0.3_ is the most invasive and challenging to fully capture using a filtering medium [52]. A filtering respirator mask is a type of personal protective equipment used to shield its user from health-harming aerosol particles [51,52,53].

Three efficiency levels and three series of filter degradation resistance are included in the nine classes of filters identified by the National Institute for Occupational Safety and Health (NIOSH). At a flow rate of 85 L/min and a pressure drop (Δp) below 343 Pa (35 mm H_2_O), the three efficiency levels are assessed at 95, 99, and 99.97%. The most penetrating particle size is typically between 0.1 and 0.3 µm. The choice of among DOP (dioctyl phthalate) liquid oil, which is significantly degrading (R or P series), and NaCl (sodium chloride salt), which is only slightly degrading to filter media (N series of filters), has developed the three degradation resistance series [45].

Europe has three types of disposable particle respirators that must comply with the European standard EN 149:2001 [54]. Like the three masks, FFP1 is primarily employed as an environmental dust mask and has an aerosol filtration of at least 80% for 0.3 μm particles. At least 94% of the FFPs are filtered by FFP2 masks, while the highest percentage of filtration is achieved by FFP3 masks. They guard against minuscule particles like asbestos with a 99% minimum filtration rate. The pressure drop during inhalation at a 95 L/min air flow rate should be less than 210 Pa for FFP1, 240 Pa for FFP2, and 300 Pa for FFP3 [54].

PBS nanofibers were successfully prepared by CS under various parameters, and the spinning parameters were also optimized, with the lowest diameter of the spun fibers being 172 nm when the concentration of the spinning solution was 7%, the rotational speed was 6000 rpm, and the size of the needle was 0.7 mm. Therefore, these parameters were chosen and productions were carried out to develop a PBS filter. The weight and thickness of the filter is 23.0 ± 0.7 g/m^2^ and 178.3 ± 70.3 µm, respectively.

**Figure 6:**
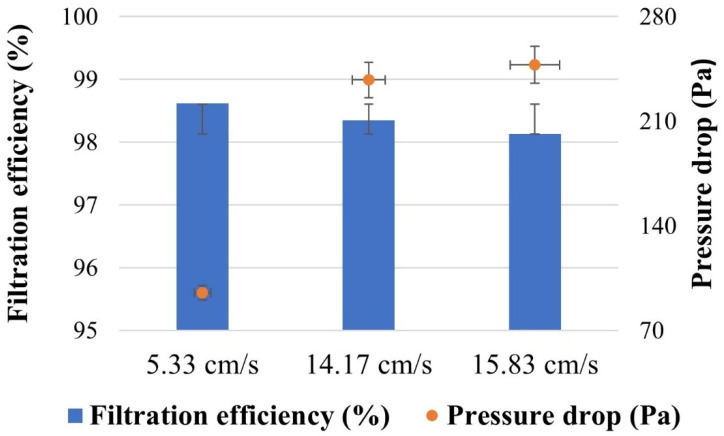
Filtration efficiency and pressure drop of the PBS filter at various face velocities.

The PBS filter was investigated as a function of face velocity, as shown in Figure 5. According to Darcy’s law of viscous resistance, the pressure drop for every fiber membrane showed a roughly linear positive connection with the filtration velocity [55]. Figure 6 depicts the filtration efficiency of the PBS filter at 5.33 cm/s, 14.17 cm/s, and 15.83 cm/s, respectively. As the face velocity increased, the filtration efficiency of the PBS filter decreased. The QF is frequently used to evaluate the overall performance of filter medium since it includes the impacts of collection efficiency and pressure drop [56,57]. The higher the QF, the more efficient the filtering and the lower the pressure drop. The QF of the PBS filter was examined as a function of face velocity, as indicated in Table 6. As the face velocity increased the QF of the PBS filter dropped. The QF values of samples were found as 0.045, 0.017, and 0.016 Pa^–1^ at face velocities of 5.33, 14.17, and 15.83 cm/s, respectively. The filtering performance of PBS filter media met N95 standards. Furthermore, it is acceptable for FFP2 filtration performance, however, the pressure drop is slightly higher.

Table 6 compares the filtration efficiency, pressure drop, and quality factor for PM_0.3_ in PBS filter media to other works. Venkataraman et al. [30] synthesized PLA filter membranes using the CS with QF 0.044 Pa^−1^, Li et al. [58] produced Chitosan/PLA nanofiber using the electrospinning with QF 0.031 Pa^−1^, and Wang et al. [55] developed PLA nanofiber by electrospinning with QF 0.064 Pa^−1^, PBS filter showed lower performance. The PBS filter was on par with or better than other filter materials, e.g., 0.11 Pa^−1^ for gelatin [44], 0.042 Pa^−1^ for zein [59], and 0.010 Pa^−1^ for cellulose acetate [60]. Notwithstanding the wide range of quality factor values, the outcome was highly promising because PBS polymer was utilized, and the quality factor was still comparable to other biodegradable polymer membranes.

### 3.4. Further Analysis of PBS Filter

**Figure 7:**
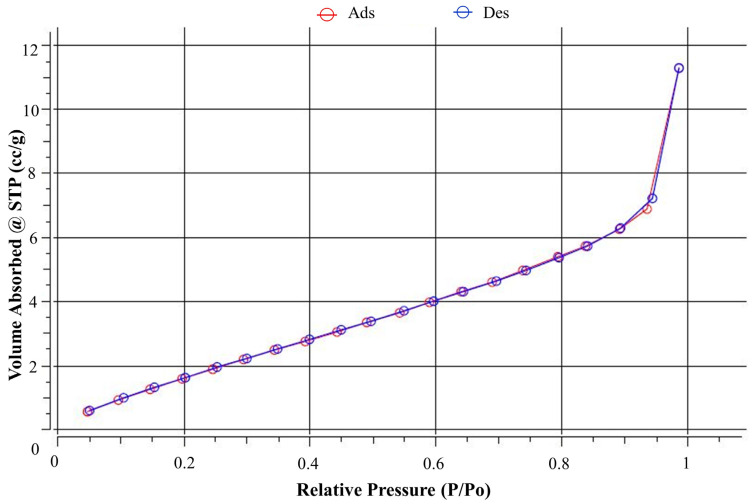
The N_2_ adsorption–desorption isotherms of PBS filter.

The surface area and pore volume of the materials produced are key features for effective filtering. The porous nature of the material was investigated utilizing nitrogen adsorption and desorption measurements on the PBS filter’s BET surface area. Table 7 summarizes the surface area, cumulative pore volume and pore diameter of the PBS filter data. The nitrogen adsorption–desorption isotherms of the PBS filter are shown in Figure 7. The findings indicated that PBS filter nanofibers have a multipoint BET surface area of 8.93 m^2^/g, indicating a significant increase in the relevant surface area when compared to other electrospun nanofiber membranes in similar studies, which found electrospun PLA, PCL, and nanofiber was 6–7.5 m^2^/g and 7.3 m^2^/g, respectively, but lower than gelation electrospun was 17.6 m^2^/g [61]. The PBS filter data is a cumulative pore volume and pore diameter of 0.016 cc/g and 3.4 nm, respectively.

Table 7 displays the mechanical parameters of the PBS filter media. As can be observed, the tensile stress of the PBS filter media was 0.10 ± 0.01 MPa with a modulus of 0.16 ± 0.12 MPa and 58.14 ± 2.13% for elongation. Cooper et al. showed that the strength and module of PBS electrospun nanofiber prepared with 10% polymer concentration is about 0.3 MPa and 0.4 MPa, respectively [18]. In addition, they also demonstrated that strength and modulus increased with increasing concentration, in this study lower results were obtained because the PBS filter was prepared at 7% concentration.

The wettability of the obtained PBS filter was tested using a contact angle device, which is a key metric for evaluating the performance of air filters. The possibility of water condensing on the pore of a polymer air filter is reduced by increasing the polymer’s hydrophobicity. Water contact with the chemical functional groups of the filters can also cause the air filter to fail to function properly [11]. The hydrophobic functional groups on the surface of the PBS nanofiber enable the polymer PBS air filter to be highly hydrophobic, and the small fiber diameter results in a smooth membrane surface and a dense fiber membrane structure. The contact angle of the samples was close to 132°, as shown in Table 7.

**Figure 8:**
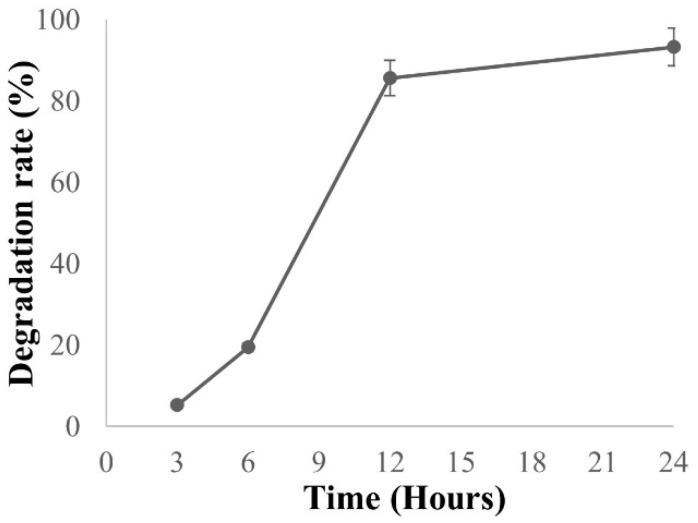
Weight loss of the PBS filter in NaOH solution for 24 h at room temperature.

The PBS filter was tested for deterioration in NaOH solution for 24 h at room temperature. Weight loss was used to calculate solubility. Figure 8 depicts the weight loss of the PBS filter. In alkaline environments, PBS is known to produce fast hydrolysis. As a result, the PBS polymer was easily converted to the oligomer and then hydrolyzed to the monomer within 24 h. Because nanofibers have a higher surface area than films or macrofibers, hydrolysis by fast absorption can be accelerated [62]. This PBS filter material was able to hydrolyze and return to nature when it was time to dispose of it.

## 4. Conclusions

The Box–Behnken design (BBD) approach was utilized for the first time in this study, to study and optimize the production of PBS nanofibers to achieve the smallest diameter with the best morphology, comprising bead-less continuous nanofibers. The minimum diameter was 172 nm at 7% solution concentration, 6000 rpm rotation speed, and 0.7 mm needle size. The polymer concentration was the most significant factor. These production parameters were utilized to create a PBS filter after they had been optimized. PBS filter met class N95 certifications with the highest filtration efficiency of ≥95% and a pressure drop of <343 Pa. BET analysis verifies the fibers’ nanoporosity, opening a wide range of potential applications. With a water contact angle of ~132°, the PBS filter media became hydrophobic. Hydrolysis enabled the disposable PBS filter to return to nature after use.

The PBS nanofiber, produced successfully via CS, would not only be a good candidate for air filtration but would also bring fresh insights into the design and development of PBS nanofiber material for diverse uses.

## Figures and Tables

**Table 1:** The experimental and predicted average fiber diameter (nm) values of PBS nanofibers are shown in the Box–Behnken 15 experimental matrix.

Run	Concentration (%)	Rotational Speed (rpm)	Needle Size (mm)	Average Fiber Diameter (nm)	
				Experimental	Predicted
R1	7	6000	0.7	172	166
R2	13	6000	0.7	286	275
R3	7	10,000	0.7	177	188
R4	13	10,000	0.7	315	321
R5	7	8000	0.6	206	201
R6	13	8000	0.6	302	303
R7	7	8000	0.8	214	214
R8	13	8000	0.8	349	354
R9	10	6000	0.6	253	264
R10	10	10,000	0.6	339	333
R11	10	6000	0.8	324	330
R12	10	10,000	0.8	340	329
R13	10	8000	0.7	277	275
R14	10	8000	0.7	264	275
R15	10	8000	0.7	283	275

**Table 2:** Factors and levels of the experimental design.

Factors	Variable Levels and Range		
	−1	0	1
Concentration (%)	7	10	13
Rotational speed (rpm)	6000	8000	10,000
Needle size (mm)	0.6	0.7	0.8

**Table 3:** The sum of squares for CS process in a sequential model.

Source	SS	df	MS	F-Value	ρ-Value	
Mean vs. Total	1.121 × 10^6^	1	1.121 × 10^6^			
Linear vs. Mean	33,489.25	3	11,163.08	8.48	0.0034	
2FI vs. Linear	1749.25	3	583.08	0.3662	0.7795	
Quadratic vs. 2FI	11,881.18	3	3960.39	23.08	0.0023	Suggested
Cubic vs. Quadratic	669.25	3	223.08	2.36	0.3110	Aliased
Residual	188.67	2	94.33			
Total	1.169 × 10^6^	15	77,946.07			

**Table 4:** Model the CS process’s summary statistics.

Linear	0.0034	0.0573	0.6157	0.3573	
2FI	0.7795	0.0438	0.5353	−0.3972	
Quadratic	0.0023	0.3110	0.9499	0.7680	Suggested
Cubic	0.3110		0.9725		Aliased

**Table 5:** ANOVA results for CS process.

Source	SS	df	MS	F-Value	ρ-Value	
Model	47,119.68	9	5235.52	30.51	0.0008	significant
A-Concentration	29,161.13	1	29,161.13	169.95	<0.0001	
B-Rotational speed	2312.00	1	2312.00	13.47	0.0144	
C-Needle size	2016.13	1	2016.13	11.75	0.0187	
AB	144.00	1	144.00	0.8392	0.4016	
AC	380.25	1	380.25	2.22	0.1967	
BC	1225.00	1	1225.00	7.14	0.0442	
A^2^	6423.08	1	6423.08	37.43	0.0017	
B^2^	76.16	1	76.16	0.4439	0.5348	
C^2^	4469.39	1	4469.39	26.05	0.0038	
Residual	857.92	5	171.58			
Lack of Fit	669.25	3	223.08	2.36	0.3110	not significant
Pure Error	188.67	2	94.33			
Cor Total	47,977.60	14				

**Table 6:** A mini summary of biodegradable nanofiber filters for air filtration for PM_0.3_.

Materials	Production Method	Air Velocity/Flow Rate	Filtration Efficiency (%)	Pressure Drop (Pa)	QF (Pa^−1^)	References
PBS	Centrifugal Spinning	5.33 cm/s	98.61	95	0.045	This study
		14.17 cm/s	98.35	238	0.017	This study
		15.83 cm/s	98.14	248	0.016	This study
PLA	Centrifugal Spinning	14.17 cm/s	99.8	140	0.044	[30]
Gelatin	Centrifugal Spinning	15.83 cm/s	-	-	0.011	[44]
PLA	Electrospinning	5.8 cm/s	99.997	165.3	0.064	[55]
Chitosan/PLA	Electrospinning	14 cm/s	98.99	147.63	0.031	[58]
Zein	Electrospinning	5.3 cm/s	99	109	0.042	[59]
Cellulose acetate	Electrospinning	85 L/min	95.33	298	0.010	[60]

**Table 7:** Mechanical and morphological properties of PBS filter.

Surface Area (m^2^/g)	Cumulative Pore Volume (cc/g)	Pore Diameter (nm)	Tensile Strength (MPa)	Young Module (MPa)	Elongation (%)	Contact Angle (°)
8.93	0.016	3.4	0.10 ± 0.01	0.16 ± 0.12	58.14 ± 2.13	131.98 ± 3.85

## Data Availability

Data are contained within the article.
